# The impact of COVID‐19 on health behaviour, well‐being, and long‐term physical health

**DOI:** 10.1111/bjhp.12520

**Published:** 2021-03-31

**Authors:** Emily McBride, Madelynne A. Arden, Angel Chater, Joseph Chilcot

**Affiliations:** ^1^ Institute of Epidemiology and Health Care University College London UK; ^2^ Centre for Behavioural Science and Applied Psychology Sheffield Hallam University UK; ^3^ Centre for Health, Wellbeing and Behaviour Change University of Bedfordshire Bedford UK; ^4^ Institute of Psychiatry, Psychology & Neuroscience King’s College London UK

**Keywords:** chronic disease, COVID‐19, health behaviour, health psychology, inequality, long‐term conditions, pandemic, prevention, well‐being

**Statement of contribution**

Emily McBride and Joseph Chilcot conceived and drafted the article. All authors contributed to the final version and approved the submission.

## Background

The COVID‐19 pandemic has led to substantial changes in population behaviour and health‐related outcomes. Approximately 10% of people who test positive for COVID‐19 experience long‐term health effects lasting for more than 12 weeks post‐infection, including persistent physical, neuropsychological, and mental health‐related symptoms (termed Long COVID; Office for National Statistics, 2020). Although outcomes are improving due to better understanding of COVID‐19 and clinical advancements, a significant proportion of people with severe disease require hospitalization and experience substantially impaired quality of life (Dennis, McGovern, Vollmer, & Mateen, 2021). There are also indirect effects on health and well‐being resulting from unemployment, trauma and stress related to the disease, bereavement, and the implementation of national and local restriction measures to control infection (Arden et al., 2020; Brooks et al., 2020; Cabarkapa, Nadjidai, Murgier, & Ng, 2020; LeRoy, Robles, Kilpela, & Garcini, 2020).

Prior to the pandemic, less than optimal engagement in preventative health behaviours (such as physical activity) and enacting risk behaviours (such as tobacco use) were estimated to account for up to 40% of the determinants of premature mortality (McGinnis, Williams‐Russo, & Knickman, 2002; Mokdad, Marks, Stroup, & Gerberding, 2004; Puterman et al., 2020). Furthermore, the combined impact of multiple preventative healthy behaviours appears to further attenuate mortality risk (Ford, Bergmann, Boeing, Li, & Capewell, 2012). Recent data from the UK Biobank reports that engagement in healthy behaviours was associated with increased life expectancy by up to 7.6 years for women and 6.3 years for men. These findings were consistent in people with and without multi‐morbidity (Chudasama et al., 2020).

Since the onset of COVID‐19, negative changes to some health behaviours have been inevitable with large segments of the population under prolonged conditions of self‐isolation, shielding, and physical distancing. Socio‐economic factors such as reduced income and job loss, as well as increased caring responsibilities (particularly for women), have posed significant barriers to engaging with health promoting behaviours, as has the impact of poorer physical and mental health. Based on UK risk factor prevalence estimates, the combined impact of physical inactivity, smoking, and obesity accounted for up to 51% of the population attributable fraction of severe COVID‐19 (Hamer, Kivimäki, Gale, & Batty, 2020). Whilst the long‐term impacts of COVID‐19 on population health and well‐being are unknown, most health experts anticipate that the effects will be profound with the most disadvantaged people in society disproportionately burdened. Here, we consider the impact of the pandemic on population health behaviours, well‐being, and long‐term physical health (including the prevention and detection of chronic disease). We also discuss the need for upstream behaviour change interventions and long‐term strategies to mitigate downstream physical and mental health consequences.

## COVID‐19 vaccination uptake

A UK population survey of 12,035 adults, between 24 November and 1 December 2020, indicated that 18% reported being hesitant (unlikely or very unlikely) to receive a COVID‐19 vaccine (Robertson et al., 2021). General mistrust in vaccines and concerns about future side effects have been listed as key barriers to optimizing population immunity (Paul, Steptoe, & Fancourt, 2020). Another UK survey between September and October 2020 reported that 11.7% of the 5,114 respondents were hesitant to receive a COVID‐19 vaccine. A large proportion of the variance (86%) in COVID‐19 vaccine hesitancy was explained by perceptions of lower collective importance, lower vaccine efficacy, potential side effects, and concerns regarding the speed of vaccine development. Vaccine hesitancy was associated with socio‐demographic factors including female gender, younger age, lower income, and ethnicity, but overall, these only explained 9.8% of the variance in the hesitation to get the vaccine (Freeman et al., 2020). In contrast, data in clinically vulnerably populations confirm high willingness for vaccination. For example, in UK patients with chronic respiratory disease, 86% of respondents wanted to receive the vaccine (Williams et al., 2020). Willingness to receive the vaccine has been associated with beliefs regarding the value to personal health, health consequences for others, concerns regarding vaccine safety, and perceptions of COVID‐19 severity (Paul et al., 2020; Williams et al., 2020). Since the vaccine was rolled out across England in priority populations, preliminary data have shown lower vaccination rates among ethnic minority groups (those with Black ethnicity displaying lowest rates), people living in areas of higher deprivation, and those with severe mental illness or learning disabilities (MacKenna et al., 2021).

## Eating behaviour and nutrition

Data from a large study of 22,374 UK adults found that one third reported changes to quantities of food consumed through the first lockdown period. Subgroups of the population were found to persistently eat more, whilst others reported eating less (particularly those who were already underweight; Herle, Smith, Bu, Steptoe, & Fancourt, 2021). Another study found that 56% of participants reported snacking more frequently (Robinson et al., 2021). Food products consumed also appear to have shifted, with a UK study finding that one third of participants reported eating less healthily than usual (McAtamney, Mantzios, Egan, & Wallis, 2021). A recent study indicated that people have consumed on average one portion of fruit and vegetables less per day than before the pandemic (Naughton et al., 2021). A potential mechanism for changes to eating behaviours may be emotion dysregulation, with those reporting changes to their eating behaviours during the pandemic also displaying greater levels of depression (Herle et al., 2021; McAtamney et al., 2021). Barriers to weight management have, for example, included reports of lower motivation to eat healthily and lower control over eating (Robinson et al., 2021).

## Physical activity and sedentary behaviour

An online UK survey of 9,190 adults found that a quarter reported lower levels of physical activity since COVID‐19 (Rogers et al., 2020). Vulnerable populations reported engaging in around half a day less of 30 min of moderate‐to‐vigorous physical activity each week through the first lockdown period (Naughton et al., 2021). Another UK survey found that 57% had either maintained or increased their levels of physical activity during lockdown. However, only a third met recommended physical activity guidelines of 150 min of moderate‐to‐vigorous physical activity per week (Spence et al., 2021). Changes in physical activity were found to be associated with components of the COM‐B model (Michie, van Stralen, & West, 2011), notably reduced physical opportunity and reflective motivation (Spence et al., 2021). Sedentary behaviour, such as prolonged periods of sitting, has also risen due to government mandates to stay at home (Wedig, Duelge, & Elmer, 2020). Sedentary behaviour is now acknowledged as an independent risk factor for poor cardiometabolic health that should be considered in addition to physical inactivity (Dempsey, Owen, Yates, Kingwell, & Dunstan, 2016). Daily sitting time increases the risk of type 2 diabetes, cardiovascular disease, and mortality (Bailey, Hewson, Champion, & Sayegh, 2019; Katzmarzyk, Church, Craig, & Bouchard, 2009), independent of physical activity levels. There is good evidence that breaking up sitting can benefit cardiometabolic health (Bailey, Mugridge, Dong, Zhang, & Chater, 2020; Brierley, Chater, Smith, & Bailey, 2019) and also improve mood and psychological well‐being (Bailey et al., 2020).

## Tobacco smoking, alcohol consumption, and substance use

A national survey of 2,254 adults in the United Kingdom indicated that one third (31%) reported drinking more alcohol through COVID‐19 than normal (King's College London, 2020). Around 24% of people with pre‐existing alcohol disorders, at high risk of relapse, reported increasing their alcohol intake (Kim et al., 2020). Data regarding substance use are more limited, with preliminary reports suggesting increased rates of drug use for substances such as cannabis, prescription benzodiazepines, and prescription opioids. Also, higher relapse rates have been observed for those in recovery from addiction (Society for the Study of Addiction, 2020).

In contrast to other behaviours, emerging evidence has suggested that tobacco quit rates and attempted quit rates have improved since the pandemic. The charity Action on Smoking and Health reported that over 1 million people had stopped smoking by July 2020, and 440,000 had attempted to quit. Increased quit rates may be due to increased motivation as a result of evidence and awareness that there is a higher risk of severe COVID‐19 and hospitalization in smokers (Action on Smoking & Health, 2020). This suggests that the pandemic may present an opportune moment for promoting tobacco control policies and smoking cessation services.

## Preventative health services and help‐seeking

Many preventative health services have seen declines in attendance and delays in help‐seeking for symptoms (Jones et al., 2020; Rees et al., 2020). For example, bowel, breast, and cervical cancer screening programmes in the United Kingdom were temporarily paused or have been operating a reduced service at different stages of the pandemic. By September 2020, an estimated 3 million people in the United Kingdom were overdue for screening (Cancer Research UK, 2020). The numbers urgently referred to secondary care with suspected cancer symptoms had also starkly declined at the height of the pandemic; though figures are now improving, they are still lower than before the pandemic. This was largely due to fewer people visiting their GP with suspected cancer symptoms but also because many GPs were reluctant to risk sending people to hospitals (Cancer Research UK, 2020). Furthermore, there have been reductions in patient engagement across general health services such as visits to Accidents and Emergency (Thornton, 2020). Some reasons reported for non‐attendance have included fear of COVID‐19 infection, not wanting to burden the health system, and practical barriers (Jo's Cervical Cancer Trust, 2020; NHS England, 2020).

## Sleep hygiene

Increased prevalence of sleep disorders through COVID‐19 has been highlighted in the research literature across different countries. Studies have examined the effect on sleep of SARS‐CoV‐2 infection and explored confounders related to isolation, quarantine, anxiety, stress, or financial losses (Partinen, 2021). It is thought that symptoms of insomnia could be related to psychosocial factors and the impact of confinement (Altena et al., 2020). A large UK study found poor quality sleep was associated with the occurrence of adverse events through the pandemic. Adverse events included, for example, illness with COVID‐19, financial difficulty, loss of paid work, problems with sourcing medicine, difficulties accessing food, and perceived threats to personal safety (Wright, Steptoe, & Fancourt, 2020).

## Mental health and well‐being

The impact of the pandemic has had a profound effect upon mental health and well‐being (Horesh, Kapel Lev‐Ari, & Hasson‐Ohayon, 2020; Lades, Laffan, Daly, & Delaney, 2020; McElroy et al., 2020; Pierce et al., 2020). Data in both the United Kingdom and the United States suggest that symptoms of psychological distress have increased during the pandemic, with people aged between 18 and 24 years showing the greatest deterioration (McGinty, Presskreischer, Han, & Barry, 2020; Pierce et al., 2020). Whilst increased psychological distress is a normal and understandable response for many given these challenging circumstances, the consequences and impact of COVID‐19 provide significant risk factors for clinically relevant anxiety and depression (Holmes et al., 2020).

Increased prevalence of social isolation and loneliness (MQ: Transforming Mental Health & the Academy of Medical Sciences, 2021) through COVID‐19 remains considerable risk factors for poorer mental health (O'Connor & Kirtley, 2018). Furthermore, social isolation and loneliness are associated with physical health outcomes, including being risk factors for type 2 diabetes (Hackett, Hudson, & Chilcot, 2020), obesity (Whisman, 2010), and incident coronary heart disease (Valtorta, Kanaan, Gilbody, Ronzi, & Hanratty, 2016). Many individuals with existing long‐term conditions (LTCs) are extremely clinically vulnerable and have been shielding for a considerable period of time, with patient surveys reporting heightened feelings of psychological distress and loneliness. Longitudinal data studies from the United States report that perceptions regarding health risks related to COVID‐19 partially explained the variability of psychological distress during the pandemic (Robinson & Daly, 2020). Furthermore, people living with LTCs have had disruptions to the accessibility and delivery of their usual care, compounded further in those needing to shield. Compared with the general population, people living with LTCs are more likely to have symptoms of depression and anxiety, which are associated with adverse clinical outcomes and events (The King's Fund, 2012). Whilst the longer‐term mental health impact of COVID‐19 on those with pre‐existing LTCs is not yet known, research and clinical interventions are needed to better understand and mitigate these effects.

## Impact of COVID‐19 on inequalities

The pandemic and COVID‐19 are disproportionately burdening low socio‐economic and black and minority ethnic (BAME) groups, as well as women and others with caring responsibilities (Chadeau‐Hyam et al., 2020; Power, 2020; Raisi‐Estabragh et al., 2020). As has been highlighted in various Government reports and the recent COVID‐19 Marmot Review, there is particular concern around rapidly widening health and social inequalities (Marmot, Allen, Goldbatt, Herd, & Morrison, 2020; UK Government, 2020). Several COVID‐19 and wider research studies have already shown that socio‐economic status, ethnicity, gender, age, and education are predictive of poorer health behaviours and health‐related outcomes (Bann et al., 2020; Marteau, Rutter, & Marmot, 2021). In addition, people in these groups display the highest risk of SARS‐CoV‐2 infection, hospitalization, and death from COVID‐19 (Marmot et al., 2020). The combined effects of worsening behavioural outcomes paired with higher risk of non‐communicable disease and severe COVID‐19 are likely to accentuate existing inequities with long‐lasting systemic effects.

## The need for tailored interventions and long‐term prevention strategies

Overall, the pandemic has been associated with adverse population trends across most health behaviours, as well as poorer mental health and well‐being outcomes. Although these trends are so far preliminary, the UK Government and international bodies anticipate long‐term systemic health and economic effects, far beyond the direct effects of COVID‐19 (UK Government, 2020). Poor health behaviours and disengagement with, or lack of access to, health services are likely to contribute to subsequent downstream physical and mental health consequences, carrying significant wider social and economic implications. Action must now be taken to mitigate the impact of COVID‐19 on health behaviours, mental well‐being, and the prevention of LTCs. Emphasis should be placed on developing, testing, and implementing cost‐effective interventions that support health behaviour change and promote health and well‐being in individuals, communities, populations, and systems. Particular consideration should be given to meeting the needs of high‐risk groups and underserved populations where inequities are already known and further anticipated. Optimal modes of delivery for interventions also need to be carefully assessed to ensure that preventative measures and support services reach all the populations they are targeting, whilst factoring in potential restrictions or access barriers in the COVID‐19 context (e.g., telephone, digital applications, online, in‐person).

Evidence from the existing health psychology and behavioural science literature can be used as a foundation and springboard to inform interventions and translate them into the COVID‐19 context. For example, already there have been systematic reviews on effective messaging which encourages vaccination uptake (Lawes‐Wickwar et al., 2021) and on psychological and behavioural responses to testing positive for other viruses (Luo, Chua, Xiong, Ho, & Ho, 2020; McBride et al., 2020). There is a wealth of established behaviour change techniques and interventions targeting health behaviours such as physical activity and healthy eating (Howlett, Trivedi, Troop, & Chater, 2019; Michie, Ashford, et al., 2011; Rhodes, Boudreau, Josefsson, & Ivarsson, 2020); tobacco smoking (Michie, Hyder, Walia, & West, 2011; Roberts, Kerr, & Smith, 2013); and alcohol consumption (Michie et al., 2012). Strategies to increase engagement with preventative health services, such as cancer screening, have been considered extensively (Camilloni et al., 2013; Sabatino et al., 2012; Tsipa et al., 2020). Similar principles can be carried forward and integrated with the proliferating and emerging COVID‐19 literature to design context‐specific tailored behaviour change and psychological interventions. These can also be used to inform the development of potential services or treatments for Long COVID.

Importantly, in tandem with evidence‐based practice, nationally recommended inclusivity and diversity frameworks such as ‘The INCLUDE Ethnicity Framework’ should be used to guide research design and intervention testing (Trial Forge; Witham et al., 2020). Guidelines produced by networks such as ‘EQUATOR’ can be used to promote transparent and accurate reporting of health research (EQUATOR Network). Patient and public involvement, which adopt representative sampling and ensure inclusion from high‐risk and underserved groups, will also be key to improving intervention quality and acceptability (Greenhalgh et al., 2019; Holmes et al., 2019).

### Conclusion

Long‐term disease prevention and detection strategies should be prioritized now that most of the UK have been living under policy restrictions for over a year, and the COVID‐19 vaccine is rolling out nationally. Cost‐effective and evidence‐based interventions that support the uptake of healthy behaviours and promote engagement with preventative health services are needed to offset indirect consequences of COVID‐19 on disease prevention and mental well‐being. Emphasis should be placed on ensuring future research and intervention development align to meet the needs of high‐risk groups and underserved populations, to help reduce widening health and social inequalities.
